# GeniePool: genomic database with corresponding annotated samples based on a cloud data lake architecture

**DOI:** 10.1093/database/baad043

**Published:** 2023-06-13

**Authors:** Noam Hadar, Grisha Weintraub, Ehud Gudes, Shlomi Dolev, Ohad S Birk

**Affiliations:** The Morris Kahn Laboratory of Human Genetics at the National Institute of Biotechnology in the Negev and Faculty of Health Sciences, Ben-Gurion University of the Negev, Beer Sheva 84105, Israel; Genetics Institute, Soroka Medical Center, Beer Sheva 84101, Israel; Department of Computer Science, Faculty of Natural Sciences, Ben Gurion University of the Negev, Beer Sheva 84105, Israel; Department of Computer Science, Faculty of Natural Sciences, Ben Gurion University of the Negev, Beer Sheva 84105, Israel; Department of Computer Science, Faculty of Natural Sciences, Ben Gurion University of the Negev, Beer Sheva 84105, Israel; The Morris Kahn Laboratory of Human Genetics at the National Institute of Biotechnology in the Negev and Faculty of Health Sciences, Ben-Gurion University of the Negev, Beer Sheva 84105, Israel; Genetics Institute, Soroka Medical Center, Beer Sheva 84101, Israel

## Abstract

In recent years, there are a huge influx of genomic data and a growing need for its phenotypic correlations, yet existing genomic databases do not allow easy storage and accessibility to the combined phenotypic–genotypic information. Freely accessible allele frequency (AF) databases, such as gnomAD, are crucial for evaluating variants but lack correlated phenotype data. The Sequence Read Archive (SRA) accumulates hundreds of thousands of next-generation sequencing (NGS) samples tagged by their submitters and various attributes. However, samples are stored in large raw format files, inaccessible for a common user. To make thousands of NGS samples and their corresponding additional attributes easily available to clinicians and researchers, we generated a pipeline that continuously downloads raw human NGS data uploaded to SRA using SRAtoolkit and preprocesses them using GATK pipeline. Data are then stored efficiently in a cloud data lake and can be accessed via a representational state transfer application programming interface (REST API) and a user-friendly website. We thus generated GeniePool, a simple and intuitive web service and API for querying NGS data from SRA with direct access to information related to each sample and related studies, providing significant advantages over existing databases for both clinical and research usages. Utilizing data lake infrastructure, we were able to generate a multi-purpose tool that can serve many clinical and research use cases. We expect users to explore the meta-data served via GeniePool both in daily clinical practice and in versatile research endeavours.

**Database URL** https://geniepool.link

## Introduction

The dramatic cost reduction in next-generation sequencing (NGS) in recent years has enabled a huge flux of human whole exome sequencing (WES) and emerging whole genome sequencing (WGS) data. However, the major databases of this large body of genomic variation are lacking in that no relevant phenotypic data are attached or can be easily accessed, hindering effective optimal use in correlating the variants with phenotypes. Assessing phenotypic impact and pathogenicity of genomic variants is a challenge being addressed in many aspects, with allele frequency (AF) being a major criterion (1). There are currently several major AF tools, such as gnomAD (2) and 1000 Genomes (3). These tools have revolutionized variant pathogenicity evaluation by enabling clinicians and researchers to assess whether a candidate mutation is present in a population in proportion to the prevalence of the corresponding investigated disease. While they provide demographic data, phenotypic attributes are absent. In many, if not most, cases, demographic data alone are insufficient. Rare diseases, for example, are apparently not so rare (4), and disease-causing mutations may still appear as seemingly non-harmful based on AF tools, including gnomAD (5). Ironically, clinicians and researchers can find a candidate mutation for a rare disease in a tool such as gnomAD in a few samples, without being able to reach out and further learn if those samples have a matching phenotype.

This problem is addressed in part by the Sequence Read Archive (6) (SRA), which contains, among others, raw sequencing data of WES and WGS from various experiments, as well as phenotypic data corresponding with the genotype. Each sample has a corresponding BioSample page containing specific data with phenotypic information (e.g. age, disease and treatment), and samples from the same study are grouped under a BioProject page which provides information regarding the study and the submitters (7). Thus, a variant in a sample from SRA is accompanied by its BioSample information, and further specific data can be obtained by contacting the submitters directly via the BioProject page. However, while raw data and sample-related information are available online through the SRA database, processing and querying the data is beyond the capabilities of non-computation-savvy individuals (8). Being a major emerging genotype–phenotype AF database, SRA is expanding rapidly. Thus, containing it requires advanced database architecture, especially when dealing with massive WGS data (9) and clinical data. Forming a framework to meet this need necessitates innovative scalability and cost-efficiency competencies which traditional methods lack compared with well-devised cloud storage architecture.

Cloud data lakes (10) are a modern approach for storing large amounts of data in a convenient and inexpensive way. The main idea is the separation of compute and storage layers. Thus, cheap cloud storage is used for storing the data, while compute engines are used for running analytics on these data in ‘on-demand’ mode. This architecture has become dominant in the industry in recent years and according to the recent studies is used at virtually all Fortune 500 enterprises (11).

To enable user-friendly accessibility to the combined genotype–sample description data in SRA, as well as practically unlimited cost-effective scalability of the rapidly expanding large body of genotypic and phenotypic data, we built GeniePool, a simple and intuitive web service and API based on a data lake architecture.

## Methods

### NGS data preprocessing

Human publicly available WES raw data from SRA are obtained using the following parameters in the SRA search bar: ‘((((illumina[Platform]) AND homo sapiens[Organism]) AND WXS[Strategy]) AND “Homo sapiens”[orgn:__txid9606] AND cluster_public[prop] AND “biomol dna”[Properties])’. The obtained table contains both SRA download accessions and corresponding BioProject and BioSample IDs. Raw sequencing data are downloaded from SRA using sratoolkit.2.11.0 ‘prefetch’ command and then extracted using ‘fastq-dump’ command, including ‘--split-files’ option if data are paired-end sequencing. Raw data are cleaned using Trimmomatic-0.39 (12) and then aligned to hg38 (UCSC version) using Picard and BWA-MEM (13) following GATK 4.2.2.0 pipeline (14) to generate VCF files using ‘HaplotypeCaller’ function. To generate parallel hg19 VCFs, we used Picard’s LiftOverVcf function using UCSC’s hg38tohg19 chain file. Variant effect annotation is done using SnpEff (15) 5.0e. This pipeline is performed using our institutional high-performance computing infrastructure at Ben-Gurion University of the Negev. Output VCF files are uploaded to the AWS S3 bucket in a gzipped format.

### Data lake architecture

GeniePool runs on AWS Cloud and consists of several building blocks. First, our variants data lake is stored in AWS S3 in Apache Parquet format. To enable efficient query of the lake, we partition it by chromosome and coordinate ranges. Second, we developed an Extract/Transform/Load (ETL) process that inserts variants from the new samples (in VCF format) into the data lake. Our ETL is implemented with Apache Spark and runs on the Amazon Web Services Elastic MapReduce (AWS EMR) platform in ‘on-demand’ mode.

### Application development

The back-end service includes a REST API that serves users’ queries over the data lake; it is written in Java and runs on the AWS Elastic Beanstalk platform. The front-end web service is written in Python and runs on AWS Elastic Beanstalk platform. Data visualization is performed using Plotly (16).

## Results

### Processed data

**Figure 1.**
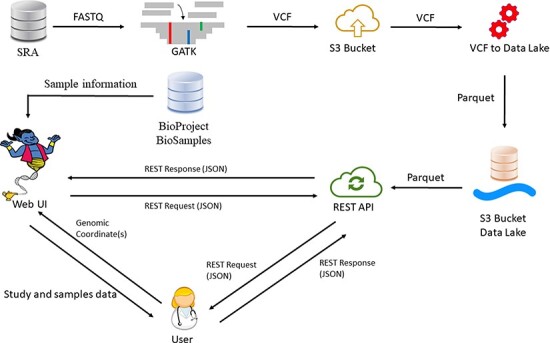
GeniePool workflow. Raw genomic NGS data from SRA are preprocessed according to GATK’s best practices and stored efficiently using the Parquet format in a cloud data lake architecture. Preprocessed data are available via either a REST API or a designated web UI accompanied by BioSample data that provide information regarding specific samples.

Our database currently houses 54 402 samples in each reference genome, originating from >970 studies. The updated number of samples is displayed in our website (https://geniepool.link) and is growing by ∼2000 samples routinely every 2 months. The total number of distinct variants is >2.2 billion for each of the reference genomes. Querying the data is accessible by either our designated REST API or our web interface (Figure 1).

### Cloud data lake efficiency and costs

At the time of submitting this manuscript, we store 1.1 TB of gzipped VCF files. The data lake, constructed from VCF files, consists of 151 K Parquet files with a total size of 171 GB. Our efficient ETL job that creates the whole data lake runs on a cluster of 30 nodes of type m5d.2xlarge with a running time of ∼2 h and a monetary cost of <10 dollars. Due to the unique architecture, storage cost of the current data lake is a ∼50 dollars per year.

### Application programming interface

GeniePool has a REST API that can receive requests including the reference genome (hg19/hg38) and genomic coordinates (e.g. 1:1000–2000). The result will be in JSON format of all variants within the specified range.

### Web–user interface

**Figure 2.**
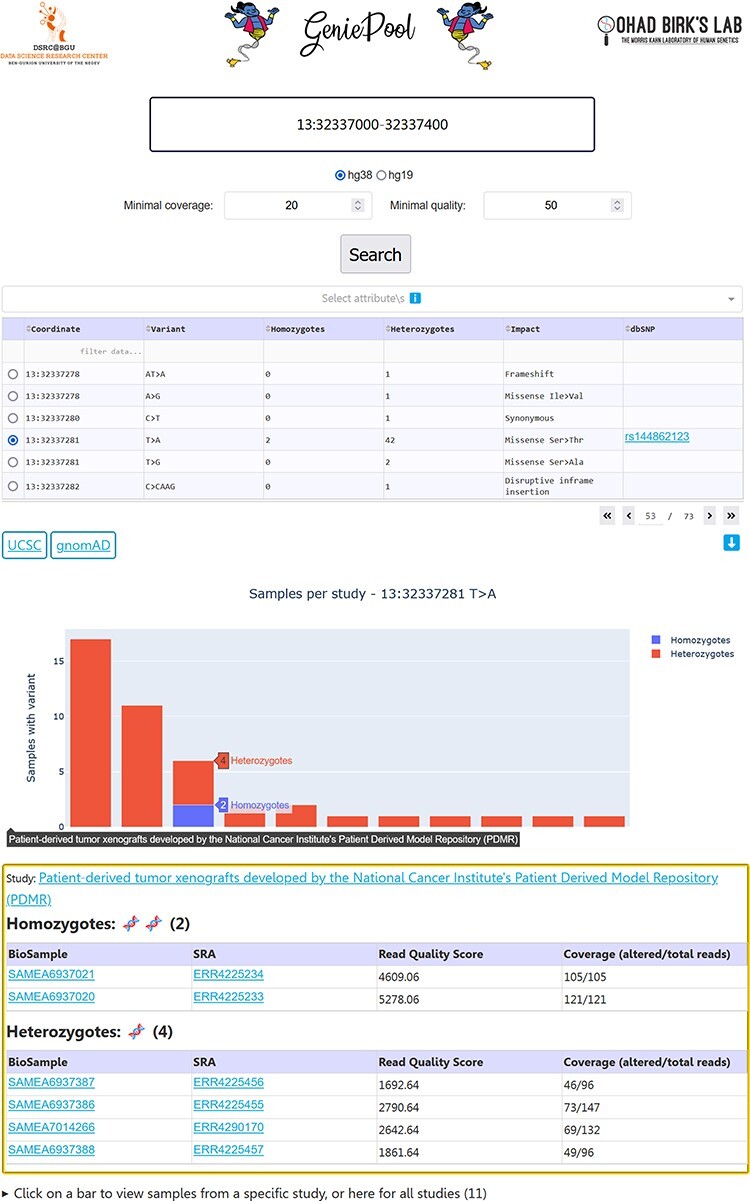
GeniePool’s UI. Genomic coordinates can be searched for variants within NGS samples from SRA. Results are displayed in a table with selectable rows. Variants can be filtered by sample attributes. Selecting a variant generates an interactive graph displaying relevant samples per study. Clicking a bar provides direct links for additional information regarding the study and each of the samples harbouring the variant.

Our simple user interface (UI) enables entering genomic coordinates in either hg38 or hg19. Variants within selected coordinates are displayed in a table with selectable rows, including dbSNP (17) IDs when available. Choosing a variant generates an interactive bar chart with a bar representing homozygotes and heterozygotes per study in BioProject who harbours the variant. Selecting a study provides links to the BioProject page and the individual BioSample pages containing data on each sample with the variant, including variant coverage and sequencing quality (Figure 2).

### GeniePool use cases

Collaboration platforms like GeneMatcher (18) have become a standard practice in clinical genomics and research. GeniePool enables a more proactive approach by enabling the user to directly search candidate variants in other NGS samples, similar to searches in platforms such as gnomAD, but then to go on to inspecting their tagged attributes. Specific attributes can be very useful, especially for excluding candidate variants, e.g. finding the variant in question in samples from patients with a different disease or even in people that were explicitly tagged as healthy individuals. In cases in which the phenotype was not tagged, users can find information outlining who uploaded the samples harbouring their variant of interest via BioSample or BioProject and contact them to advance their case. This feature can be very important when considering late-onset disease. Our group has already used this methodology multiple times. For one, we study the Israeli Bedouin population extensively, an ethnicity that is not significantly represented in databases such as gnomAD. GeniePool made available >1200 Qatari exomes from a specific study on the Qatari population (19). This is extremely useful for us for in silico filtration of variants that are rare globally, but not locally. We have also contacted other groups in cases where a variant was found in just a few samples, asking them to check if a certain phenotype was overlooked in their patients. This can be proved to be a fertile strategy for initiating collaborations.

## Discussion

GeniePool has been designed to enable cost-effective massive storage and easy querying of phenotypic and genotypic data of a practically unlimited number of samples. It is a novel application for evaluating variants based on their prevalence in the SRA dataset of human NGS. However, unlike other AF databases, rather than only providing information about AF for given coordinates, GeniePool delivers specific sample and study data.

In our hands, GeniePool has now become an effective tool for further analysis of candidate disease-causing genomic variants. In fact, several of our research projects have already undergone revision due to finding samples of other groups with several studied candidate variants that had documented phenotypes not resembling those of our studied families. It should be noted that many other variants were already ruled out during prior variant analysis using standard tools. Thus, GeniePool enabled ruling out further variants that could not have been ruled out by other AF databases, saving precious time and resources. We are now developing an in-house variant analysis tool which has, among other features, a direct link to GeniePool regarding each variant.

Our greatest challenges are to fully utilize both massive numbers of NGS samples and their corresponding information provided by BioProject and BioSample. Regarding these two databanks, their data do not have a consistent form and are mostly in free text. Our data lake approach is ideal for confronting both tasks. We plan on enriching GeniePool’s functionality by supporting queries not only on genomic coordinates but also on meta-data derived from the published papers associated with the imported samples using artificial intelligence (AI) techniques. Also, we plan to have all publicly available WES samples in a matter of months. The following step will be adding WGS samples, which devour much more computational resources and storage, challenges well met by our cloud data lake architecture. Finally, we will adjust our pipeline to automatically update the database with each newly uploaded WES or WGS data onto SRA.

Until we integrate BioProject and BioSample automatically into search queries, it should be emphasized that GeniePool links its user to these databanks. It is important to note that even if specific data concerning a sample or a project are not noted, the BioProject page can lead directly to the submitters of the data. This itself has the potential to initiate collaborations or in-depth queries regarding phenotypic significance of specific variants.

Introducing GeniePool to its intended audiences, including researchers and clinicians, presents a significant challenge due to its superficial similarity to gnomAD. Both databases provide the ability to search for information about specific variants in thousands of samples. However, the use cases of the two databases are distinct. gnomAD provides AF and ethnicity information per variant, while GeniePool, being composed of various studies, cannot provide such data accurately. Furthermore, samples in gnomAD are entirely anonymous, and further information about individuals harbouring interesting variants is not available. This limitation is where GeniePool sets itself apart by providing comprehensive and specific data on samples from previous studies. It is worth noting that this paradigm shift may not be immediately intuitive for the target audience, which is accustomed to using NGS databases mainly for querying allele frequencies, a capability that has significantly impacted clinical genomics. Nonetheless, GeniePool is not intended to compete with databases such as gnomAD, but rather, it is a complementary software that addresses other research and clinical needs.

At the moment, GeniePool serves as an extension of SRA, BioProject and BioSample by combining processed data from each source and storing them in a data lake. This project demonstrates the advantage of using data lakes for genetic and clinical data integration. While SRA contains raw sequencing data, usually in FASTQ format, GeniePool’s data lake stores preprocessed VCF data without the intermediate BAM files generated in the GATK pipeline. We are aware of the fact that ideally, users would like to analyse both FASTQ and BAM files in various methods that cannot be used on the preprocessed VCF file, such as using tools for finding viral DNA (20), structural variants (21) and mobile element insertions (22). We are planning on expanding the types of files and tools that will be accessible via GeniePool, knowing that building the project on top of a data lake makes our plans feasible for dealing for versatile types of data and algorithms in a scalable and cost-effective way. Currently, GeniePool focuses on VCF data as a step towards the realization of the ideal utilization of data lakes for making genomic data accessible.

SRA contains massive amount of raw RNA-sequencing (RNAseq) data of various experiments from multiple biological material and under multiple conditions, truly unpolished diamonds for researchers. Nevertheless, the common clinician and researcher do not possess the skills to preprocess such data—the exact same situation regarding WES and WGS data that GeniePool solves by preprocessing the raw data beforehand and making it accessible with now available algorithms that can deal with extended amount of RNAseq samples (23). The same basic idea behind GeniePool can serve as the basis for making accessible the continuously uploaded raw RNAseq data uploaded onto SRA, with data lakes being the preferred way to efficiently confront the challenge of managing SRA’s constantly growing data loads.

It should be noted that GeniePool cannot include SRA samples that are also listed in dbGaP (24, 25) (the database of genotypes and phenotypes) because they require specific permission for access. GeniePool thus only includes samples that were uploaded to SRA as public data and therefore require no permission for download. While there are about five times more controlled access samples than publicly available ones, the latter have continued to grow by the thousands yearly (Supplementary Figure S1), providing GeniePool the potential for continuous expansion.

The scalability of the data lake architecture is also suitable for managing sequencing data along with other heavy-duty medical data, such as radiological imaging files. Such data can provide the means to utilize various algorithms integrate genetic and other heavy-duty types of medical data (26).

Regarding reference genomes used by GeniePool, hg38 is used for raw data alignment, and then liftOver from the hg38 files is further used to support hg19 as well. The new CHM13 reference genome (27) (also known as T2T) will also be supported once it becomes more prevalent in either research or clinical settings. To maximize the utilization potential of CHM13, generating its variant data will be done using realigning raw data to it and not by using liftOver.

GeniePool has been set up as a genotype–phenotype database, enabling advanced queries for deciphering multi-genic diseases, creating collaborations and a tool that can facilitate many research endeavours. Its data versatility encouraged us to make it available as soon as possible. We expect that it will enable users to answer scientific questions in both straightforward as well as creative manners, utilizing its combined easily interrogated combined genotypic–phenotypic vast information.

## Supplementary Material

baad043_Supp

## Data Availability

GeniePool source code is available in the GitHub repository (https://github.com/geniepool). GeniePool web UI is available at: https://GeniePool.link REST API is available using: http://api.geniepool.link/rest/index/$reference/$coordinates (with hg38/hg19 for reference and chr:start-end for coordinates, e.g. http://api.geniepool.link/rest/index/hg38/1:12345789-123456798). Further information and instructions regarding the API are available under the frequently asked questions (FAQ) section of the web UI.
