# Supplementary figures and images for: GeniePool: genomic database with corresponding annotated samples based on a cloud data lake architecture
Source: Database (Oxford). 2023 Jun 13;2023:baad043. doi: 10.1093/database/baad043 (PMC10263466; doi:10.1093/database/baad043)

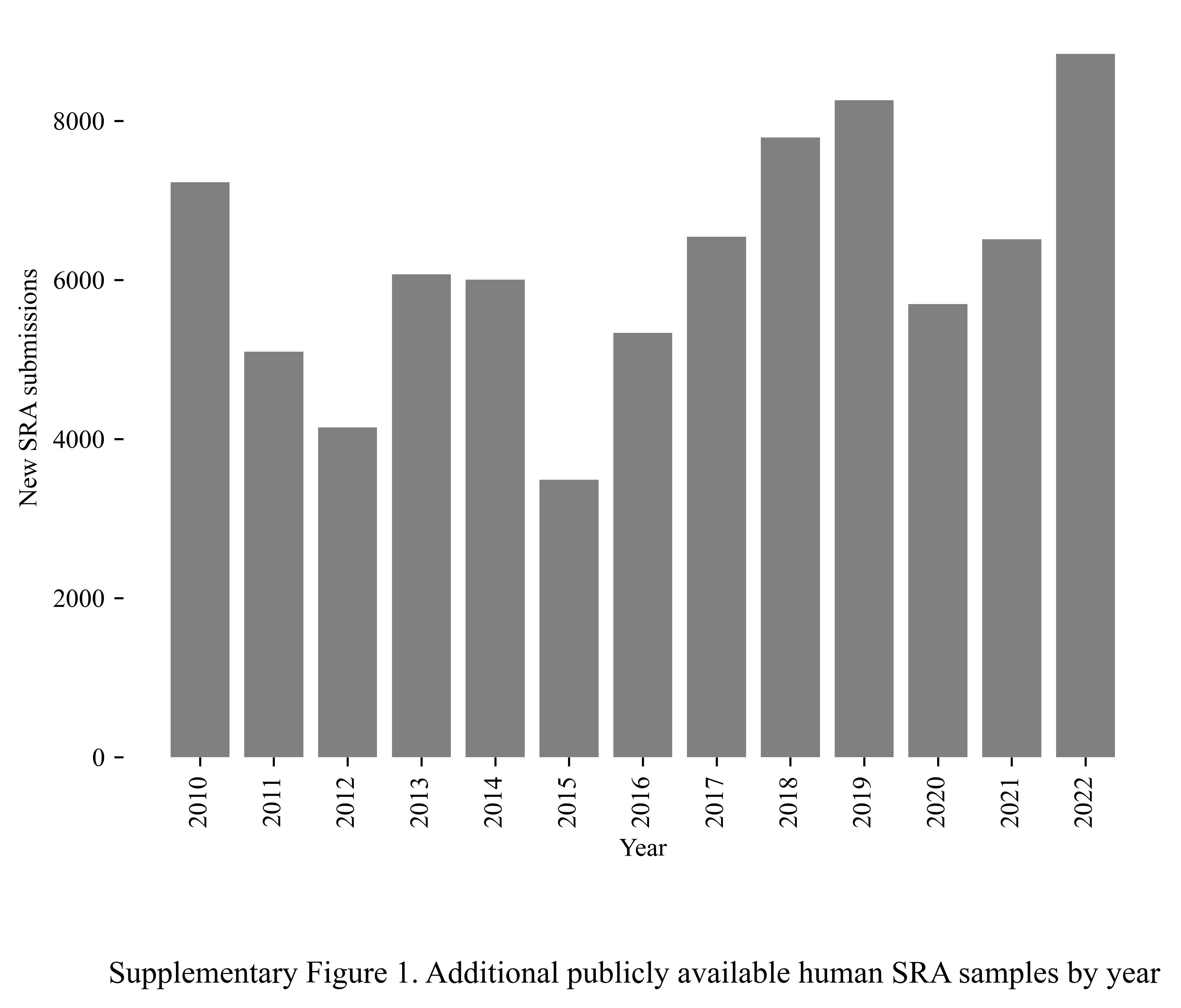

Supplement: baad043_Supp [file baad043_supp.zip › suppl_data/Supplementary Figure 1.tif]
